# Resection of a giant right coronary artery aneurysm and reconstruction with a saphenous vein graft: a 20-year follow-up—case report

**DOI:** 10.1093/ehjcr/ytae357

**Published:** 2024-09-03

**Authors:** Joshua Halyckyj-Smith, David Rose

**Affiliations:** University of Manchester, Royal Preston Hospital, Lancashire Teaching Hospitals Trust, Sharoe Green Ln, Fulwood, Preston PR2 9HT, UK; Lancashire Cardiac Centre, Blackpool Victoria Hospital, Whinney Heys Rd, Blackpool FY3 8NP, UK; Lancashire Cardiac Centre, Blackpool Victoria Hospital, Whinney Heys Rd, Blackpool FY3 8NP, UK

**Keywords:** Case report, Coronary artery aneurysm, Resection and reconstruction, CT angiography

## Abstract

**Background:**

Coronary artery aneurysms (CAAs) are uncommon and can cause complications such as thrombosis, vessel rupture, or distal embolization. Rarely, CAAs are classified as ‘giant’, although the defining diameter is debated. The predominant cause of CAAs is atherosclerotic disease. Independently, CAAs constitute an estimated 5-year survival of 71%.

**Case summary:**

We report the case of a 56-year-old female who presented 20 years ago with a chest infection when a murmur was auscultated on examination. Subsequently, a coronary angiogram was performed, demonstrating an extensive aneurysm of the right coronary artery (RCA). The aneurysmal segment of the RCA was resected, and a length of saphenous vein was utilized in its reconstruction. Twenty years later, the patient re-presented with dyspnoea, indicating repeat investigations; coronary angiography demonstrated a vein graft 20 years post-reconstruction that is almost indistinguishable from a native RCA.

**Discussion:**

The optimal management strategy for CAAs is debatable, and there are no clear guidelines. However, surgical management is generally preferred in cases of GCAAs, which was also the case for this patient. This reconstruction procedure, involving resection of the aneurysmal segment of the RCA and reconstruction with a saphenous vein graft, proved to be a durable and reliable approach, with the saphenous vein graft remaining patent for over 20 years. The 20-year follow-up provides valuable insight into the long-term durability of surgical intervention, allowing for comprehensive assessment of the durability and reliability of this procedure.

**Learning points**

The case report provides valuable insights into the management of giant coronary artery aneurysms and its long-term success. The main learning points derived from this report include the following:

- Long-term success of the procedure, both in the durability of surgical approach and long-term patency of saphenous vein graft.
- Detailed documentation of the surgical approach will allow for a greater understanding of the techniques utilized, contributing to the knowledge base for managing *similar* cases. However, the detailed description of the surgical approach emphasizes the importance of tailoring intervention to the individual characteristics of the patient’s anatomy and comorbidities. This requires a holistic, multidisciplinary approach.

## Introduction

Coronary artery aneurysms (CAAs) are rare, with an incidence of around 4.9%.^1^ A CAA is defined as a *focal* pathological dilation of the coronary artery >1.5 times the diameter of the adjacent portion of the vessel.^2^ However, the consensus for defining a *giant* coronary artery aneurysm (GCAA) is unclear; the defining diameter ranges from 8 mm^3^ to in excess of 20 mm.^4^ Specific guidelines regarding the management of CAAs have not been developed, reflecting the morphological variation of disease. The objective of this case report is to present a rare case of GCAA with its *long-term follow-up*, to highlight the durability of the surgical approach and saphenous vein graft patency, and to reinforce the importance of an individualized and multidisciplinary approach.

## Summary figure

**Figure:**
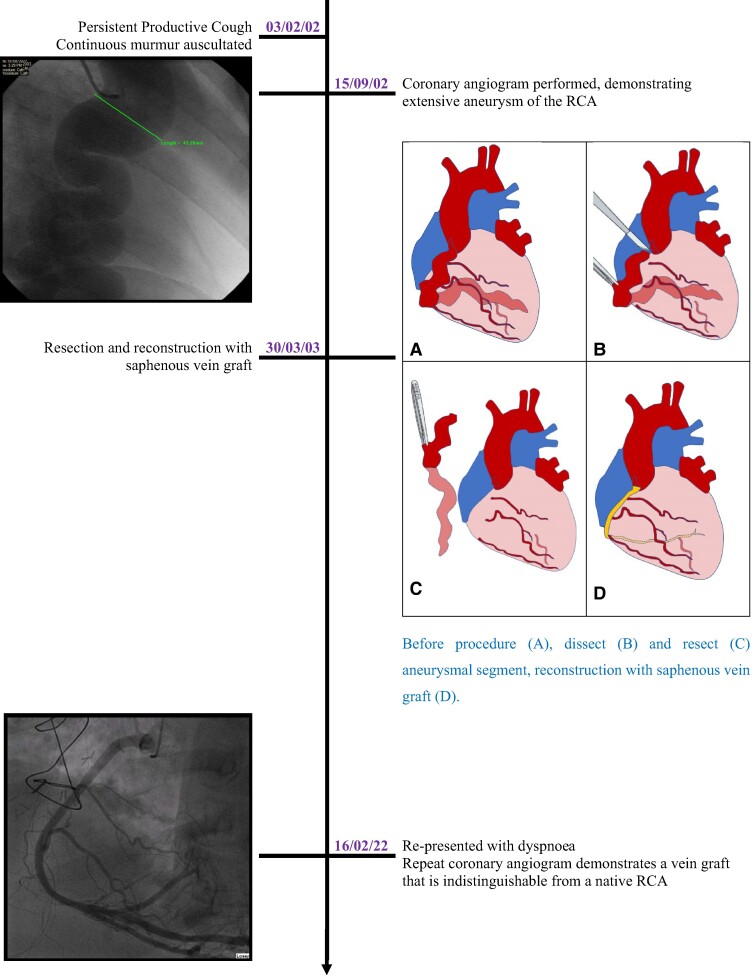
Timeline of events, demonstrating resection and reconstruction to be a durable and reliable approach, with the vein graft remaining patent for over 20 years.

## Case presentation

**Figure 1:**
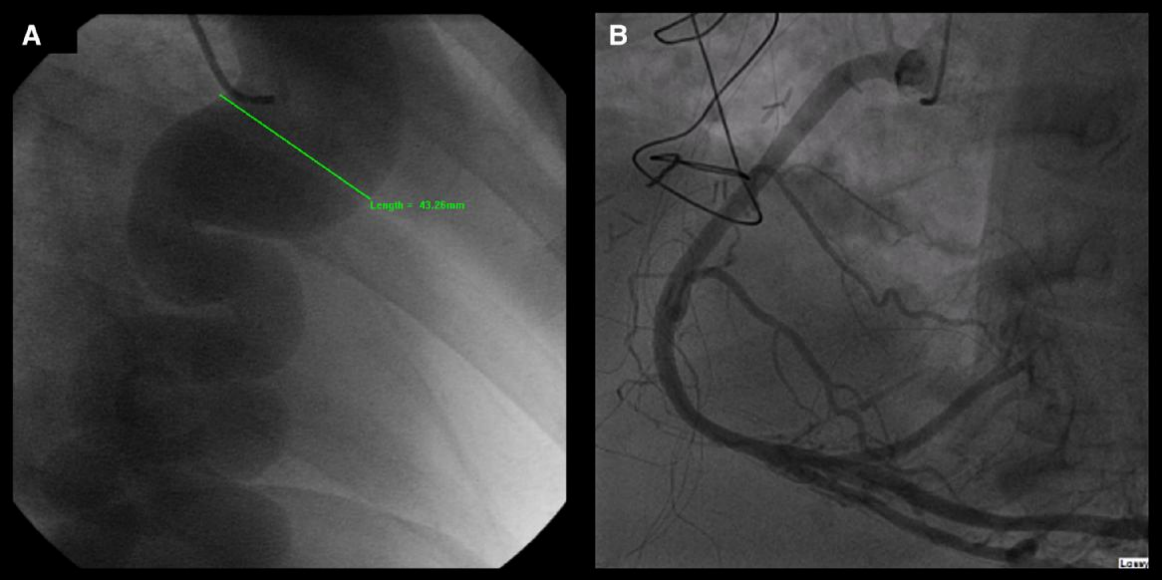
Coronary angiogram: pre-operative (*A*) and at 20-year follow-up (*B*).

A 56-year-old female, with a background of hypertension and depression, who was a never-smoker, presented to her general practitioner in 2001 with dyspnoea and a progressive, productive cough. An incidental continuous murmur was auscultated on examination, and she was referred to secondary care for investigation. Echocardiographic data at this time were unavailable, but coronary angiography revealed a right coronary artery (RCA) that was aneurysmal from 1 cm distal to its origin through to its fistulation with the coronary sinus. At its largest diameter, the aneurysm measured around 43 mm (*Figure 1A*). A fistulous connection between the AV circumflex into the pulmonary artery and a leash of small fistulae arising from the LAD were also identified. The patient was diagnosed with a right CAA and multiple coronary fistulae and underwent a routine admission for resection and reconstruction of a right CAA.

**Figure 2:**
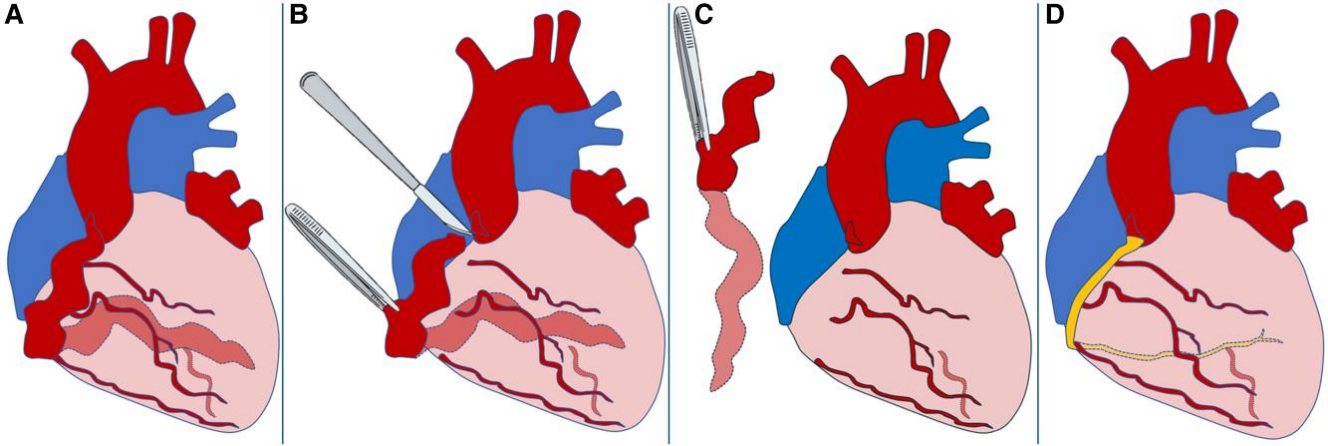
Before procedure (*A*), dissection (*B*) and resection (*C*) of the aneurysmal segment, and reconstruction with a saphenous vein graft (*D*).

The aneurysmal segment of the RCA was resected, and a length of saphenous vein was anastomosed, utilizing end-to-side technique, to its most distal identifiable branch. The vein graft was then sequentially anastomosed to a series of four side-to-side anastomoses with isolated buttons of RCA branches, before its proximal anastomosis to the proximal segment of the native RCA. The procedure is depicted in *Figure 2*.

Post-operatively, the patient was transferred to the care of the cardiac surgical unit. She remained haemodynamically stable, requiring a minimal dose of noradrenaline to maintain satisfactory blood pressure. Her recovery was complicated by the development of atrial fibrillation, but amiodarone was commenced with good efficacy. Her recovery was further complicated by the development of a pleural effusion and small pneumothorax; a drain was inserted and was left *in situ* for 24 h before removal. The post-removal chest X-ray demonstrated satisfactory resolution.

On the seventh post-operative day, the patient was mobilizing well and deemed fit and stable enough for discharge. She was discharged with aspirin 300 mg o.d., senna two tablets o.d., Maxolon 10 mg TDS, amiodarone (200 mg TDS for 1 week, followed by 200 mg b.d. for 1 week, followed by 200 mg o.d. for 1 week), and paracetamol 1 g QDS.

Following discharge, she made a good recovery, with minimal dyspnoea, unlimited walking distance, and no symptoms suggestive of congestive heart failure. Long-term risk factor modification was achieved with long-term aspirin, beta-blocker, angiotensin-converting enzyme inhibitors, digoxin, and rivaroxaban therapy, but the exact treatment protocol was unavailable for publication.

Twenty years later, the patient re-presented with dyspnoea, prompting repeat investigations. *Figure 1B* demonstrates that the vein graft used for the reconstruction 20 years ago was patent, with no evidence of stenosis or occlusion; the saphenous vein graft was almost indistinguishable from a native RCA.

**Figure 3:**
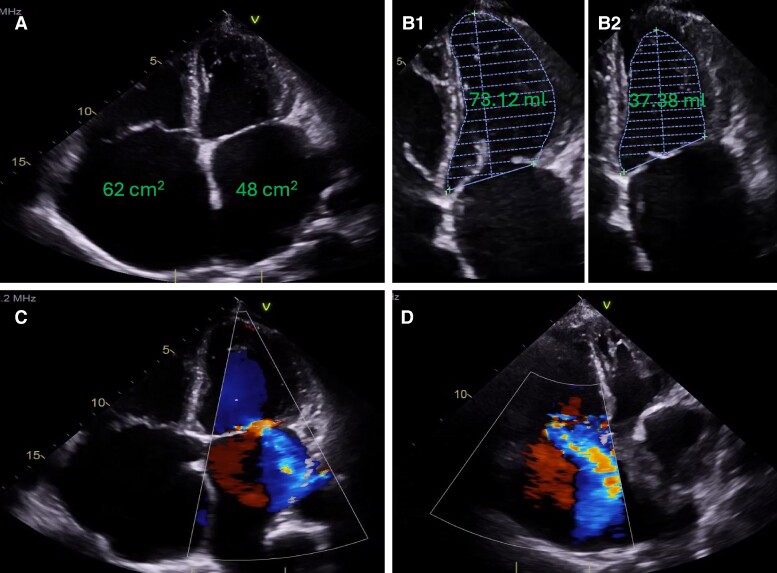
Transoesophageal echocardiogram at 20-year follow-up. Bi-atrial dilatation with atrial volumes (*A*), left ventricular function [end-diastolic volume (*B.1*), end-systolic volume (*B.2*)], mitral regurgitation (*C*), and tricuspid regurgitation (*D*).

While the electrocardiogram (ECG) was documented to demonstrate no significant findings apart from their long-standing history of atrial fibrillation (ECG unavailable), the transoesophageal echocardiogram (*Figure 3*) alluded to the cause of her symptoms: gross bi-atrial dilatation (*Figure 3A*), impaired left ventricular function (ejection fraction in the region of 45%; *Figure 3B*) particularly in view of severe mitral regurgitation (*Figure 3C*), and severe tricuspid regurgitation (*Figure 3D*). Therefore, she was reviewed at the cardiac multi-disciplinary team meeting with view of mitral valve replacement. However, due to her level of frailty and the high-risk nature of a redo procedure, it was decided, in conjunction with the patient’s wishes, that the proposed surgical intervention was unsuitable. Therefore, optimal medical management was continued.

## Discussion

### Strengths and weaknesses

The 20-year follow-up provides valuable insight into the long-term durability of surgical intervention, allowing for comprehensive assessment of the durability and reliability of this procedure and saphenous vein graft patency.

While this case report offers valuable insight into the long-term success of surgical management in the context of GCAAs, it is essential to recognize the inherent limitations associated with a single-case study. As this report focuses on a single patient, the generalizability of the findings to a broader population is limited due to the morphological variation of GCAAs, and caution should be exercised in extrapolating the results to other patients. While the 20-year follow-up is valuable, it may introduce potential bias due to selective patient follow-up.

### Pathology

Coronary artery aneurysms most commonly affect the RCA, followed by the left anterior descending and circumflex coronary arteries.^5^ The predominant driver of CAA formation is proposed to be atherosclerotic disease, a disease *process* which develops over many years.^6^ However, the pathophysiological mechanism remains unclear.^6^ Potential explanations include degradation of the wall integrity with a reduction in tolerance to the intraluminal pressures. Histological studies support this pathophysiological mechanism, demonstrating overall thinning of the tunica media, with hyalinized connective tissue replacing the normal smooth muscle cells and elastic fibres that are usually present to provide strength and stability to the coronary artery.^5^

While most patients with a CAA have atherosclerotic disease, only a minority of patients with atherosclerotic disease present with a CAA; CAA formation is the product of a multivariate disease process. Potential genetic drivers of aneurysm formation include variants of chromosome 9p21.3,^4^ in addition to polymorphisms in the matrix metalloproteinase 3 gene.^4^ Further notable risk factors include congenital destruction of the tunica media, connective tissue disorders such as Kawasaki disease and Marfan’s syndrome, and various forms of vasculitis.^7^

### Diagnosis

Coronary artery aneurysms are typically an incidental finding at coronary angiography. However, when symptoms manifest, the spectrum of symptoms experienced depends on the underlying cause and morphology of the aneurysm. As the primary driver of CAA formation is atherosclerotic disease, the predominant clinical presentation is comparable to that of typical coronary artery disease.^8^ The irregularity of the internal surface of the aneurysmal wall reduces flow velocity and induces turbulent flow, predisposing the induvial to thrombus formation and consequential embolization.^8^ This sequela results in angina pectoris, dyspnoea, acute coronary syndrome, or even sudden death.^9^ Rarely, a systolic murmur is audible over the precordium.^10^ In contrast, GCAAs are typically symptomatic and may even present with compression effects that mimic a mediastinal mass or cardiac malignancy,^9^ such as superior vena cava syndrome.^11^ Other presentations include haemopericardium, cardiac tamponade, and congestive heart failure.^11^

Coronary artery aneurysm can be diagnosed via many techniques, both invasive and non-invasive. However, coronary angiography remains the gold standard as it provides information about the shape, size, location, and number of aneurysms,^12^ which is invaluable in devising management plan that is tailored to the individuals’ anatomy. Other diagnostic investigations include echocardiography, computerized tomography, and magnetic resonance imaging.

### Management

Specific guidelines regarding the management of CAAs have not been developed, reflecting the morphological variation of the disease. A multidisciplinary approach, involving cardiac surgeons, interventional cardiologists, and radiologists, ensures comprehensive patient evaluation and optimal treatment planning. Currently, management options comprise medical, percutaneous, and surgical approaches that should be tailored to individuals’ anatomy and comorbidity status.

Medical therapy involves aggressive risk factor modification and includes guideline-directed medical therapy to reduce atherosclerotic risk factors.^13^ Furthermore, if thrombosis is a prominent concern, anticoagulation should be considered.^13^ Specific medical therapy should also be considered if there is the presence of an underlying precipitating condition.^7^

Percutaneous approaches aim to exclude the aneurysm from the coronary circulation. Covered stents are the recommended choice where anatomy permits, despite concerns of deliverability, risk of re-stenosis and thrombosis, and accidental occlusion of side branches with subsequent myocardial ischaemia.^10^ An alternative percutaneous approach is aneurysmal coiling.^10^

Surgical management is usually an alternative therapy for those with CAAs who are not suitable for percutaneous intervention. However, surgical management is generally accepted as the preferred option in cases of GCAAs.^11^ Surgical approaches include resection and reconstruction, proximal and/or distal ligation, thrombectomy, and aneurysmectomy with or without bypass grafting.^14^ The procedure utilized should be tailored to the patient’s anatomy and comorbidity status, and should include guideline-directed medical therapy to reduce atherosclerotic risk factors.^13^

### Prognosis

Giant coronary artery aneurysms are rare and can cause complications such as thrombosis, rupture, or distal embolization. Giant coronary artery aneurysms are complex entities; thus, long-term prognosis is controversial. However, as an independent risk factor, patients have an estimated 5-year survival of 71%.^15^

## Conclusion

This case report describes a middle-aged woman with a GCAA that was managed with a surgical approach. The specific approach outlined involved resection of the aneurysmal segment and reconstruction with a saphenous vein graft. Alternative approaches are available and outlined in the text. However, this case highlights the importance of a tailored and multidisciplinary approach in achieving long-term success in the management of patients with GCAAs.

*This procedure proved to be a durable and reliable approach, with the vein graft remaining patent for over 20 years.*

## Data Availability

The data underlying this article are available in the article and in its online supplementary material.

## Lead author biography

Joshua Halyckyj-Smith is a final-year medical student at the University of Manchester. He is a representative of the Society for Cardiothoracic Surgery in Great Britain and Ireland through his role on the INSINC committee. His current interests lie in adult cardiac surgery, but he hopes to further explore his interests in congenital cardiac surgery.

## Notes

**Consent:** The authors confirm that verbal consent for submission and publication of this case report, including images and associated text, has been obtained from the patient in line with COPE guidance.

**Funding:** University of Manchester School of Medicine: The University of Manchester Faculty of Biology Medicine and Health, open access agreement with Oxford University Press.
